# A pilot study: effectiveness of local injection of autologous platelet-rich plasma in treating women with stress urinary incontinence

**DOI:** 10.1038/s41598-020-80598-2

**Published:** 2021-01-15

**Authors:** Cheng-Yu Long, Kun-Ling Lin, Chin-Ru Shen, Chin-Ru Ker, Yi-Yin Liu, Zi-Xi Loo, Hui-Hua Hsiao, Yung-Chin Lee

**Affiliations:** 1 Department of Obstetrics and Gynecology, Kaohsiung Municipal Siaogang Hospital, Kaohsiung Medical University, Kaohsiung, Taiwan; 2 Department of Obstetrics and Gynecology, Kaohsiung Medical University Hospital, Kaohsiung Medical University, Kaohsiung, Taiwan; 3 Graduate Institutes of Clinical Medicine, College of Medicine, Kaohsiung Medical University, Kaohsiung, Taiwan; 4 Department of Internal Medicine, Kaohsiung Medical University Hospital, Kaohsiung Medical University, Kaohsiung, Taiwan; 5 Department of Urology, Kaohsiung Municipal Siaogang Hospital, Kaohsiung Medical University, 482 Shan-ming Road, Kaohsiung, 812 Taiwan; 6 Department of Urology, College of Medicine, Kaohsiung Medical University, Kaohsiung, Taiwan; 7 Department of Urology, Kaohsiung Medical University Hospital, Kaohsiung, Taiwan; 8 Regenerative Medicine and Cell Therapy Research Center, Kaohsiung Medical University, Kaohsiung, Taiwan

**Keywords:** Medical research, Urology

## Abstract

The study aims to evaluate the effectiveness of local injection of autologous platelet rich plasma (A-PRP) as a treatment for women suffering from stress urinary incontinence (SUI). In a prospective intervention study, twenty consecutive women suffering from SUI were treated with A-PRP injection at anterior vaginal wall where mid-urethra locates. Self-reported questionnaires were used to measure pre-treatment, 1 month and 6 months post-treatment symptom severity. Secondary outcomes of sexual function and treatment effect sorted by age were analyzed with valid statistical methods. A-PRP is effective in relieving SUI symptoms at both 1 month and 6 months post-treatment without significant adverse reactions reported. It seems to have a trend that treatment success rate with cured and improved symptoms was slightly higher in the younger group, although it did not reach statistical significance (*P* = 0.07). No significant changes in sexual function before and after the treatment were reported by the patients. This pilot study is the first to report A-PRP treatment effect for SUI in women. The result suggested that A-PRP is a considerable treatment option for mild to moderate SUI cases. It also opens up further research opportunities for A-PRP’s clinical applications.

## Introduction

Platelet is rich in various kinds of growth factors and cytokines that promote soft tissue healing. Insulin-like growth factor (IGF), epidermal growth factor (EGF), basic fibroblast growth factor (bFGF), platelet-derived growth factor (PDGF), transforming growth factors-beta (TGF-β), vascular endothelial growth factors (VEGF), connective tissue growth factor (CTGF) hepatocyte growth factor (HGF) and interleukin 8 (IL-8) are only a few to name^1^. Each has its role in enhancing cell migration, cell recruitment, cell replication, extracellular matrix scaffolding, tissue regeneration and neo-angiogenesis. These processes are activated upon stimulation by exposure to thrombin, calcium or collagen in vivo^2^. Collectively, they repair damaged tissues and rejuvenate aged cells. Autologous platelet-rich plasma (A-PRP) is synthesized from patients’ own blood after concentration via centrifugation. The autologous nature meant satisfying safety profile for reduced immune reactions, as long as the preparation is handled with care and good sterile techniques.

A-PRP has long-standing history and good outcomes in sports medicine, particularly in treating tendonitis, arthritis, ligament sprains and tears. It is effective in reducing injury pain, fast healing and quick return to regular activities. Other fields with evidence-based applications include dentistry, dermatology^2^, sports medicine^3^, cardiac surgery, pediatric surgery, urology^4^, plastic surgery and ophthalmology^5^. Utilization of A-PRP in the field of obstetrics and gynecology dated back as early as 2007 by the Fanning J et al., who investigated direct application to operated wounds in total abdominal hysterectomy, laparoscopy-assisted vaginal hysterectomy and urogynecology surgeries. They found significantly reduced procedure related pain as early as post-operation day 1^6^. However, no available literatures published so far have demonstrated potential therapeutic effect of A-PRP in treating women with stress urinary incontinence (SUI).

SUI is a bothersome gynecology problem all around the world, with an estimated prevalence of 40% in adult women^7^. Birth trauma, aging, obesity and estrogen deprivation are well-known risk factors. Kunkle and colleague reported approximately 13.12 billion US dollars were spent on SUI, including disposable diapers, laundry, dry cleaning, and sanitary pads^8^. A variety of treatment modalities is currently available: lifestyle modification and pelvic floor muscle exercise might be effective for mild degree of SUI symptom; electrostimulation, biofeedback, and extracorporeal magnetic innervation are non-invasive symptom-control methods.

Anti-incontinence surgeries, such as mid-urethral tapes and colposuspension are effective and durable^9^. Each method has its strengths and limitations that should be adopted according to individual condition, characteristics, disease severity, and economic considerations to reach a shared decision making between the healthcare provider and patients. This study aims to assess A-PRP as an alternative treatment option for the treatment of SUI. Reviewing the literature, this is the first report that demonstrates clinical outcomes of A-PRP application in stress-incontinent women.

## Results

Among the 20 patients enrolled, the average age was 44.5 years old with averaged parity of 1.6 times, body mass index 22.7 mg/m^2^. Five or 25% of them are menopause. Prior to the PRP treatment, the average pad test was 5.8 g; one (5%) reported mild symptom while 12 (60%) and 7 (35%) reported moderate and severe to very severe diseases, respectively by International Consultation on Incontinence Questionnaire-Short Form (ICIQ-SF) score. All patients followed for at least 6 months (Table 1). Treatment efficacy assessed by ICIQ-SF, Urogenital Distress Inventory (UDI-6), Incontinence Impact Questionnaire (IIQ-7) and Overactive Bladder Symptom Scores (OABSS) showed significant incontinence improvement at both 1 month and 6 months post treatment, but not by Pelvic Organ Prolapse Distress Inventory 6 (POPDI-6) (Table 2).

**Table 1:** Demographic data (n = 20) are given as mean ± standard deviation or n(%).

Mean age (years)	44.5 ± 9.1
Mean parity	1.6 ± 0.5
Mean BMI (kg/m^2^)	22.7 ± 6.3
Pad test	5.8 ± 3.6
Menopause	5 (25.0)
**SUI grade (ICIQ-SF)**	
Mild	1 (5.0)
Modetate	12 (60.0)
Severe and very severe	7 (35.0)
Follow up (months)	6

BMI, body mass index, Values are expressed as mean ± standard deviation or numbers; SUI grade according to ICIQ-SF: slight (1–5), moderate (6–12), severe (13–18) and very severe (19–21).

**Table 2:** Questionnaire results at baseline and 1, 6 months post-treatment.

N = 20	Baseline	1 months post-Tx	6 months post-Tx	*P* value*	
				1 month	6 months
ICIQ-SF	11 (6–18)	6 (0–17)	4 (0–16)	0.012*	0.002*
UDI-6	33.3 (17–72)	22.2 (0–78)	17 (6–72)	0.005*	0.004*
IIQ-7	23.8 (5–90)	4.8 (0–86)	4.8 (0–86)	0.001*	0.016*
OABSS	6 (0–12)	3 (0–12)	4 (0–12)	0.034*	0.229
POPDI-6	4 (0–13)	2 (0–15)	2 (0–14)	0.254	0.232

Tx treatment, ICIQ-SF International Consultation on Incontinence Questionnaire-Short Form, UDI-6 Urogenital Distress Inventory, IIQ-7 Incontinence Impact Questionnaire, OABSS Overactive Bladder Symptom Scores, POPDI-6 Pelvic Organ Prolapse Distress Inventory 6.

Values are expressed as median (range).

*Statistical significance; Wilcoxon signed rank test.

**Figure 1:**
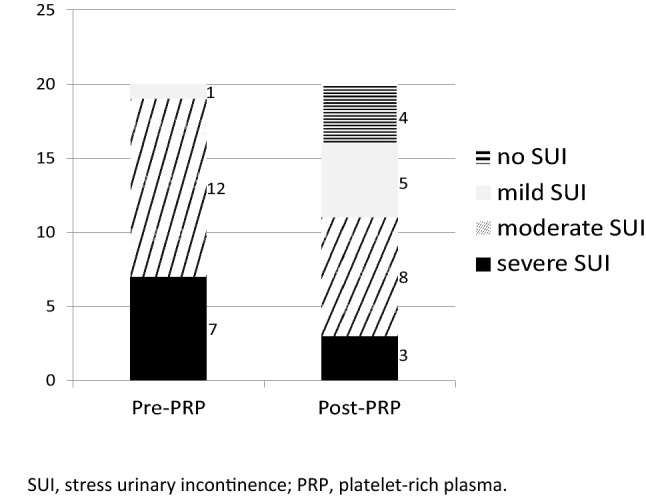
Changes in grades of stress urinary incontinence following treatment of PRP injection.

Changes in grades of SUI following treatment of PRP injection were shown in Fig. 1. These women were then sorted by age, with the cut-off value at 40 years old to investigate age as a factor for treatment efficacy. The result showed no significance between the characteristics of the two groups in terms of mean body mass index, underlying diabetes mellitus, hypertension, history of hysterectomy or history of mid-urethral sling. Only parity number was significantly higher for the older group. However, it seems to have a trend that treatment success rate with cured and improved symptoms was slightly higher in the younger group (75%) compared to that of the older group (50%), although it did not reach statistical significance (*P* = 0.07). The treatment efficacy is also demonstrated by disease severity distribution pre-PRP and 6 months post-PRP (Table 3), with a shift of the majority reporting moderate and severe diseases to moderate and milder diseases. No adverse reactions were reported.

**Table 3:** Treatment efficacy of PRP injection.

Pre-op	Post-op						
	Cure	Mild	Moderate	Severe	Improved	Unchanged/worse	Efficacy
Mild (n = 1)	1	0	0	0	1	0	1/1 (100%)
Moderate (n = 12)	3	3	5	1	6	6	6/12 (50%)
Severe (n = 7)	0	2	3	2	5	2	5/7 (71.4%)
All (n = 20)	4	5	8	3	12	8	12/20 (60%)
95% CI					95% CI [3.89, 9.01]	95% CI [− 2.59, 0.15]	

PRP, platelet-rich plasma; Pre-op, preoperative; Post-op, postoperative; CI, confidence interval.

Only 8 women completed the urodynmaic studies before and 6 months after intervention. Residual urine and bladder volume at first sensation to void increased significantly after injection of PRP. All other urodynamic parameters showed no significant differences following treatment (Table 4). The influence of PRP injection on sexual function was investigated by Female Sexual Function Index (FSFI) questionnaire. No significant changes before and after the treatment were reported by the patients, neither for the total score or for each component (i.e. desire, arousal, lubrication, orgasm, satisfaction and dyspareunia) (Table 5). Further analysis by the factor of age revealed there were no in-between group differences (Table 6).

**Table 4:** Urodynamic changes before and 6 months after PRP treatment.

Parameters	Pre-op (n = 8)	Post-op (n = 8)	*P* value #
Qmax (ml/s)	29.8 ± 5.4	33.7 ± 4.1	0.08
RU (ml)	9.7 ± 5.1	46.7 ± 13.4	0.028 §
FS (ml)	135.8 ± 42.5	163.8 ± 17.0	0.001 §
MCC(ml)	428.0 + 21.5	408.8 ± 41.1	0.17
Pdet (cmH_2_O)	24.5 ± 5.4	23.8 ± 5.8	0.52
FUL (mm)	24.8 ± 6.2	25.4 ± 6.8	0.22
MUCP (cmH_2_O)	65.5 ± 9.8	54.1 ± 21.5	0.14

Data are given as n(%) or mean ± standard deviation.

PRP, platelet-rich plasma; DO, detrusor overactivity; Qmax, maximum flow rate; RU, residual urine; FS, first sensation to void; MCC, maximum cystometric capacity; Pdet, detrusor pressure at peak flow; FUL, functional urethral length; MUCP, maximum urethral closure pressure.

^#^Paired t-test; § significant significance.

**Table 5:** Changes Female Sexual Function Index (FSFI) scores at baseline and 6 months post-treatment with PRP injection.

	Pre-treatment	6 months post-treatment	*P* value
FSFI total scores	17.9 ± 10.2	20.3 ± 11.0	0.25
Desire (1, 2)	2.7 ± 1.3	2.7 ± 1.2	0.79
Arousal (3–6)	2.6 ± 1.8	2.9 ± 0.9	0.46
Lubrication (7–10)	3.2 ± 2.1	3.5 ± 2.1	0.34
Orgasm (11–13)	3.0 ± 1.9	3.4 ± 2.1	0.35
Satisfaction (14–16)	3.2 ± 2.0	3.8 ± 2.3	0.13
Pain (17–19)	3.2 ± 2.0	3.9 ± 2.4	0.11

Data are given as median (range) or mean ± standard deviation.

Paired’s t-test.

**Table 6:** Changes in scores of Female Sexual Function Index in both groups before and 6 months after treatment.

Domains	Age < 40 (n = 8)			Age > 40 (n = 12)			
	Pre-op	Post-op	*P* value*	Pre-op	Post-op	*P* value*	Intergroup *P* value^
Sexual desire	2.6 ± 1.4	2.9 ± 1.2^	0.03**	2.8 ± 1.3	2.3 ± 1.1^	0.014**	P^a^
Sexual arousal	2.5 ± 1.7	3.2 ± 1.2^	0.19	3.7 ± 1.0	3.0 ± 1.3^	0.029**	P^b^
Lubrication	3.5 ± 1.8	4.3 ± 1.1^	0.30	4.0 ± 1.5	3.6 ± 2.0^	0.23	P^c^
Orgasm	2.8 ± 1.5	3.6 ± 1.6^	0.27	4.2 ± 1.3	4.1 ± 1.7^	0.71	P^d^
Satisfaction	3.1 ± 1.7	4.2 ± 1.7^	0.22	4.4 ± 1.3	4.5 ± 1.9^	0.77	P^e^
Dyspareunia	3.8 ± 1.7	4.7 ± 1.2^	0.24	3.4 ± 1.6	3.7 ± 2.3^	0.39	P^f^
Total scores	18.3 ± 8.5	22.8 ± 6.7^	0.21	24.3 ± 6.1	24.1 ± 6.7^	0.89	P^g^

Data are given as mean ± standard deviation.

Pre-op, preoperatively; Post-op, postoperatively. *Paired *t*-test; ^Student’s *t*-test; **Statistical significance. P^a^ = 0.72, P^b^ = 0.59, P^c^ = 0.48, P^d^ = 0.13, P^e^ = 0.20, P^f^ = 0.22, P^g^ = 0.59.

## Discussion

The current study demonstrates A-PRP is effective in treating women with SUI for as long as 6 months post treatment. The outcome is evidenced by multiple self-reported questionnaires before, 1 month and 6 months after the treatment. ICIQ-SF, UDI-6, IIQ-7 and OABSS questions all revealed significant and lasting effectiveness, while POPDI-6 showed a trend of improved symptom scores although not reaching statistical significance (Table 2). Figure 1 further illustrated a shift of disease severity distribution to milder diseases after PRP treatment. According to the integral theory, the most important factor in cases of female SUI is a pubourethral ligament defect^10^. Nikolopoulos and colleagues have advocated the plausibility of A-PRP in restoring pubourethral ligament integrity to treat SUI in as early as 2016^11^. He promoted his hypothesis by laying out various animal models that proved the rejuvenating abilities of A-PRP compositions, such as VEGF, IGF-1, PGDF, HGF, TGF-β and FGF. Injecting bulking agents to provide mechanical support of urethral, thereby storing normal pelvic anatomy and reducing urethral hypermobility, is not new in treating SUI. A-PRP serves a superior agent than previously employed paraffin, bovine collagen, polydimethylsiloxane, polyacrylamide gel, and hyaluronic acids^1^, for its autologous nature and minimal, if any, allergic reactions. A-PRP is not only biocompatible, durable and non-migratory; its reparative ability can repair damaged ligaments and potentially prolong treatment effectiveness.

A secondary analysis of this study observed a superior treatment outcome in the patient group younger than 40 years old. It is postulated that being younger might pose better rejuvenating abilities, and thus better treatment effect of A-PRP. Also, aged patients are likely to be impacted with additional variables such as more parity number, menopausal state, more severe SUI symptoms and more underlying systemic conditions such as diabetes mellitus and hypertension, history of hysterectomy, abdominal surgeries and previous anti-continence treatments. The reparative ability in aged people’s plasma might be reduced is another hypothesis to be tested. These potential confounding factors are demonstrated by a trend in our small study groups, although not reaching statistical significance. Further study with greater patient number and variable-adjusted analysis is required to validate the assertion. If proven, these factors would be very helpful for patient selection that will benefit the most from PRP treatment.

Sexual function is intimately related to urinary incontinence, thus warranting a secondary analysis in the treatment of SUI with A-PRP. The current study utilizes FSFI questionnaires pre- and post-PRP treatment but failed to reveal significantly improved composite sexual function score, regardless of length of follow up (Table 2). However, when examining each domain of FSFI, an improved sexual desire was noted in both age groups (Table 3). This signifies how stress urinary incontinence impacts the patient’s self-image, confidence and fear of embarrassment when it comes to sexual functions. Therefore, an improvement in SUI symptoms readily enhances sexual desire. Interestingly, our finding is in contrary to Runels and colleagues’ 2014 report, which demonstrated significantly improved FSFI performance in total scores, desire, arousal, lubrication and orgasm domains in 11 women receiving PRP for sexual disorders^12^. The authors also reported prolonged prolong arousal, ejaculatory orgasm, spontaneous orgasm in younger women as side effects that resolve in 2 weeks without treatment. The observation is not revealed by our study, which is mainly attributed to disparity in injection sites. Runels’ group, who specifically aimed at treating sexual dysfunctions, injected A-PRP at both clitoris and a spot of anterior vaginal wall most distal from the urinary bladder. Our study that aimed at treating urinary incontinence had a different injection site. Cultural difference where sexual topics are not as openly discussed in Asian populations might also contribute to the differential results.

PRP it has only gained more attention in the field of obstetrics and gynecology in recent years. Targeted conditions encompass symptomatic ectopic cervix^13^, lichen sclerosis at vulvovaginal^14^, vesicovaginal fistula^15^, pelvic organ prolapse^16,17^, ovarian function rejuvenation^18^, endometrial receptivity^19^, female sexual dysfunction^12^, membrane sealant in preterm pre-labor ruptured membrane^20^ and cesarean section wound aesthetics^21^. We ever reported that the treatment efficacy for the vaginal Er:YAG laser for SUI at 6-month follow-up was 75.5%^22^. Promising impact of PRP and carbon dioxide laser for SUI was also noted recently^23^.

However, the effect of PRP alone on SUI was rarely reported.

The current study is the first report of A-PRP application on women with SUI, with treatment outcomes demonstrated by before and after treatment questionnaires scores. The results are analyzed objectively with valid statistical methods. An intermediate follow-up time of 6 months was investigated. Secondary analysis of impacts of age and sexual functions were also reported. Limitations of the work included small sample size and lack of a controlled group. Future work is encouraged to incorporate a randomized controlled trials with longer follow-up period. Comparative study with head-to-head comparisons to other bulking agents is plausible. Other potential areas to investigate may include A-PRP as a preventative role at the time of pelvic floor structure insult, as an adjuvant modality in combination with corrective surgery or other conservative treatments, determination of its lowest effective dose, repeat treatment intervals if necessary and long-term efficacy.

## Conclusion

Local injection of autologous platelet rich plasma seems safe with somewhat satisfactory response in treating female SUI both at 1 month and 6 months post treatment. It appears to have a trend that younger women have better treatment outcome, and larger sample sizes might shed more light upon this effect. Yet how long the treatment effect could sustain remains unknown. This innovated intervention could be an alternative treatment for SUI but awaits further explorations.

## Material and methods

From June 2018 to November 2018, females with SUI (involuntary loss of urine on effort or physical exertion or on sneezing or coughing), age over 20 years old, the platelet counts within normal limit (150 k–450 k/uL) and Prothrombin Time (PT) was normal, were offered the innovative treatment of autologous platelet-rich plasma (A-PRP) injections. The study employed a prospective interventional design. A total number of 20 consecutive patients consented to enter the trial with full awareness of the experimental nature and treatment process without compensations in any form. The treatment utilizes autologous material that poses minimal adverse reactions to the patients.

**Figure 2:**
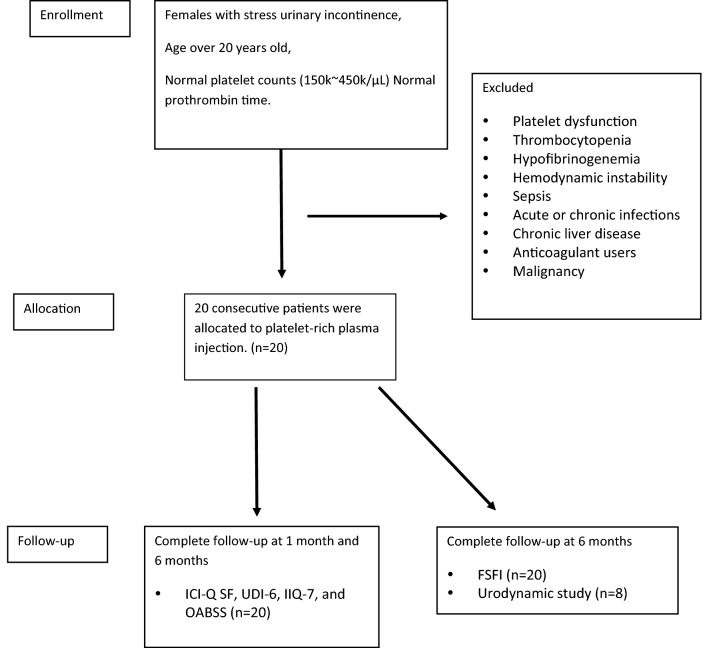
The clinical trial flowchart for platelet-rich plasma injection.

The Institutional Review Board (IRB) Committee of Kaohsiung Medical niversity Hospital approved this research (IRB Number: KMUHIRB-F (I)-20170048) and confirmed that all methods were performed in accordance with the relevant guidelines and regulations. This clinical trial was registered in a publically accessible primary register that participates in the WHO International Clinical Trial Registry Platform with Clinical trial registration number ID: NCT04279210) and date of registration (21/02/2020). All procedures involving human participants were in accordance with the ethical standards of Institutional Review Board of Kaohsiung Medical University Hospital and with the 1964 Helsinki declaration and its later amendments or comparable ethical standards. Patients with the following conditions were excluded from the study: known platelet dysfunction, critical thrombocytopenia, hypofibrinogenemia, hemodynamic instability, sepsis, acute or chronic infections, chronic liver disease, anti-coagulant users and known malignancy (Fig. 2). They did not receive concurrent treatment for SUI during the study period.

**Figure 3:**
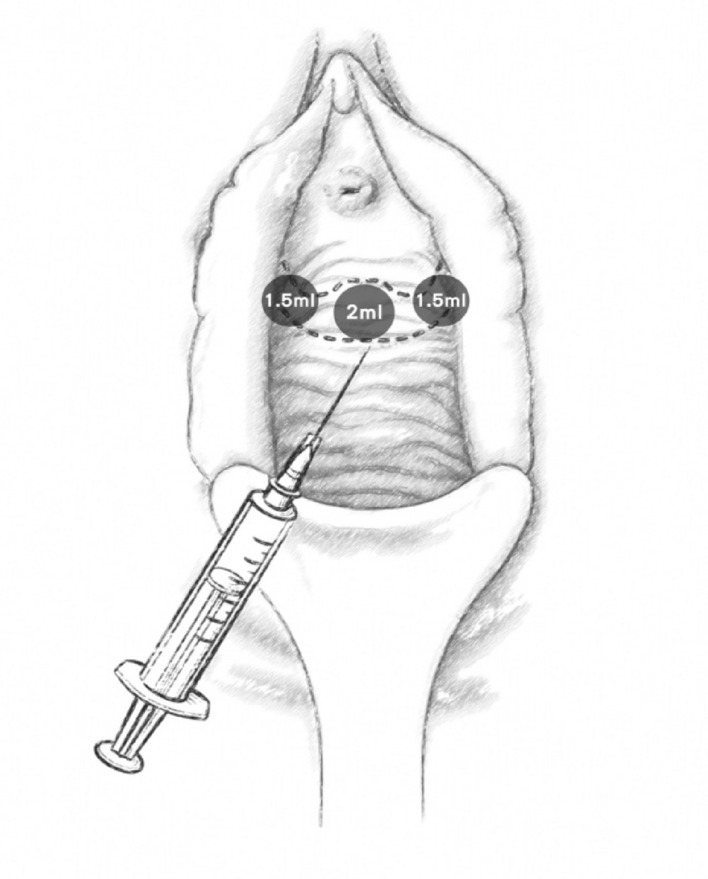
Diagram of the injection site and depth over anterior vaginal mucosa.

The study employed commercialized PRP kit from RegenKit (Regenlab, Le Mont-sur-Lausanne, Switzerland). PRP was prepared according to the standardized procedures instructed by the kit. Two tubes preloaded with anticoagulant additive were used to collect individual patient’s whole blood, 10 mL each. The content was mixed by inverting the tubes gently 4–5 times. The tubes were centrifuged at 3400 rpm for 15 min. After centrifugation, three-layered content was noted with platelet pellet, separating gel and red blood cells from top to bottom in order. Platelet pellet was remixed with the supernatant by inverting the tubes gently for 5–10 times. The supernatant then was collected in a 5-mL Luer-Lock syringe. The yielded volume was approximately 5 mL from each tube. The Regen system was specifically designed to produce APRP with a platelet concentration of 1.6X. With a 27-gauge needle, PRP was injected into the anterior vaginal mucosa around the patient’s mid-urethra, which was approximately 1 cm below the urethra meatus with a depth about 1.5 cm. Two mL underneath mid-urethra and 1.5 mL for each side of urethra (Fig. 3). No anesthesia was used in this procedure. Monthly treatment was given for 3 consecutive months.

Before enrollment, the patients signed informed consent for participation. The primary outcome was the degree of SUI relief, while the secondary outcome assesses sexual function. Urodynmaic studies were scheduled for them before and 6 months after treatment. Questionnaires that assess the severity of their urinary incontinence and sexual dysfunction were distributed. Specifically, they were asked to fill out OABSS, UDI-6, IIQ-7, ICIQ-SF, POPDI-6 and FSFI questionnaires. ICIQ-SF was the primary tool for assessing SUI severity, which was categorized into slight, moderate, severe and very severe according to the total points obtained after answering the questionnaire. The cut-off values were 1–5, 6–12, 13–18 and 19–21 points for slight, moderate, severe and very severe, respectively^24^. Women were deemed for cure if they felt no complaint of SUI after procedure. Symptom improvement was recognized with downgrading of the ICIQ-SF results. Data were collected before, 1 month and 6 months post-treatment. Statistical analysis was performed using Student’s t test and paired t-test. A difference was considered statistically significant when *P* < 0.05.

## References

[1] Andia, E., Rubio-Azpeitia, J., Martin, I. & Abate, M: Current concepts and translational uses of platelet rich plasma biotechnology. In *Biotechnology* (ed. Ekinci, D.) (InTech, 2015). 10.5772/59954. https://www.intechopen.com/books/biotechnology/current-concepts-and-translational-uses-of-plateletrich-plasma-biotechnology.

[2] Alves R, Grimalt R (2018). A review of platelet-rich plasma: history, biology, mechanism of action, and classification. Skin Appendage disord..

[3] Guevara-Alvarez A, Schmitt A, Russell RP, Imhoff AB, Buchmann S (2014). Growth factor delivery vehicles for tendon injuries: mesenchymal stem cells and platelet rich plasma. Muscles Ligaments Tendons J..

[4] Matz EL, Pearlman AM, Terlecki RP (2018). Safety and feasibility of platelet rich fibrin matrix injections for treatment of common urologic conditions. Investig. Clin. Urol..

[5] Moutray T (2018). Different lasers and techniques for proliferative diabetic retinopathy. Cochrane Database Syst. Rev..

[6] Fanning J (2007). Phase I/II prospective trial of autologous platelet tissue graft in gynecologic surgery. J. Min. Invas. Gynecol..

[7] Hunskaar, S., Burigio, K., Diokno, A. C., Herzog, A. R., Hjalmas, K. & Lapitan, M. C. Epidemiology and natural history of urinary incontinence. In *Incontinence: 2nd International Consultation on Incontinence. Recommendations of the International Scientific Committee: The Evaluation and Treatment of Urinary Incontinence*. Paris, 1–3 July 2001 (ed. Abrams, P., Cardozo, L., Khoury, S. & Wein, A.) Medline: 20304 (Health Publication Ltd, Plymouth, UK, 2002).

[8] Kunkle CM (2015). Cost utility analysis of urethral bulking agents versus midurethral sling in stress urinary incontinence. Female Pelvic Med. Reconstr. Surg..

[9] Capobianco G (2018). Management of female stress urinary incontinence: a care pathway and update. Maturitas.

[10] Petros PE, Ulmsten UI (1990). An integral theory of female urinary incontinence, experimental and clinical considerations. Acta Obstet. Gynecol. Scand. Suppl..

[11] Nikolopoulos KI, Pergialiotis V, Perrea D, Doumouchtsis SK (2016). Restoration of the pubourethral ligament with platelet rich plasma for the treatment of stress urinary incontinence. Med. Hypotheses.

[12] Runels C, Melnick H, Debourbon E, Roy L (2014). A pilot study of the effect of localized injections of autologous platelet rich plasma (PRP) for the treatment of female sexual dysfunction. J. Women’s Health Care.

[13] Hua X (2012). Using platelet-rich plasma for the treatment of symptomatic cervical ectopy. Int. J. Gynaecol. Obstet. Off. Organ Int. Fed. Gynaecol. Obstet..

[14] Behnia-Willison F (2016). Use of platelet-rich plasma for vulvovaginal autoimmune conditions like lichen sclerosus. Plast. Reconstr. Surg. Glob. Open.

[15] Bodner-Adler B, Hanzal E, Pablik E, Koelbl H, Bodner K (2017). Management of vesicovaginal fistulas (VVFs) in women following benign gynaecologic surgery: a systematic review and meta-analysis. PLoS ONE.

[16] Chrysanthopoulou EL (2017). Platelet rich plasma as a minimally invasive approach to uterine prolapse. Med. Hypotheses.

[17] Einarsson JI, Jonsdottir K, Mandle R (2009). Use of autologous platelet gel in female pelvic organ prolapse surgery: a feasibility study. J. Min. Invas. Gynecol..

[18] White YA (2012). Oocyte formation by mitotically active germ cells purified from ovaries of reproductive-age women. Nat. Med..

[19] Colombo GVL (2017). Use of platelet rich plasma in human infertility. J. Biol. Regul. Homeost. Agents.

[20] Lewi L (2009). In vitro evaluation of the ability of platelet-rich plasma to seal an iatrogenic fetal membrane defect. Prenat. Diagn..

[21] Tehranian A (2016). Application of autologous platelet-rich plasma (PRP) on wound healing after caesarean section in high-risk patients. Iran. Red Crescent Med. J..

[22] Lin KL, Chou SH, Long CY (2019). Effect of Er:YAG laser for women with stress urinary incontinence. Biomed. Res. Int..

[23] Behnia-Willison F, Nguyen TTT, Norbury AJ, Mohamadi B, Salvatore S, Lam A (2019). Promising impact of platelet rich plasma and carbon dioxide laser for stress urinary incontinence. Eur. J. Obstet. Gynecol. Reprod. Biol. X.

[24] Klovning A, Avery K, Sandvik H, Hunskaar S (2009). Comparison of two questionnaires for assessing the severity of urinary incontinence: the ICIQ-UI SF versus the incontinence severity index. Neurourol. Urodyn..

